# What stresses adolescents? A qualitative study on perceptions of stress, stressors and coping mechanisms among urban adolescents in India

**DOI:** 10.12688/wellcomeopenres.16818.1

**Published:** 2021-05-11

**Authors:** TK Nagabharana, Shama Joseph, Azeez Rizwana, Murali Krishna, Mary Barker, Caroline Fall, Kalyanaraman Kumaran, GV Krishnaveni

**Affiliations:** 1 Epidemiology Research Unit, CSI Holdsworth Memorial Hospital, MYSORE, Karnataka, 570022, India; 2 Foundation for Research and Advocacy in Mental Health (FRAMe), MYSORE, Karnataka, 570022, India; 3 MRC Lifecourse Epidemiology Unit, University of Southampton, Southampton, Hampshire, SO16 6YD, UK

**Keywords:** Adolescents, stress, coping, India, family conflict, academic pressure; qualitative research

## Abstract

**Background:** In India, stress levels are increasing steadily among youth. We aimed to explore the factors that contribute to psychological stress and coping strategies among adolescents in Mysore, India to inform the development of an intervention.

**Methods:** We recruited 16 volunteers in Mysore, India including 6 younger (12-15 years; 3 girls) and 10 older adolescents/ young adults (17-25 years; 5 girls) using a purposive sampling technique. Older adolescents were recruited from ongoing birth cohort study, and the younger adolescents by word-of-mouth from the community. Individual in-depth interviews were carried out based on a semi-structured interview guide comprising open-ended questions. The interviews were analysed to derive themes and emerging constructs related to stress and coping strategies.

**Results:** **Adolescents generally perceived stress in their daily lives. Family conflicts and academic pressures were the main triggers for increased stress. Issues around peer relationships, and social position were also important contributors. Adolescents reported that they had robust coping strategies. These included stress release through rationalising and acceptance of the situation, distraction activities, spirituality, and self-comforting methods. However, they felt the need for further support from their family, and the society in general. In particular they expressed the need for a space to share their concerns and obtain guidance through healthy discussions with adults.

**Conclusions:** Our study indicates that adolescents in India are exposed to a wide range of stressors in their daily lives. The conflict between ‘traditional’ society’s opinions of what adolescents should do and the new age adolescents’ aspirations for autonomy to find ‘informed’ solutions for their issues may hinder the stress management efforts. Moving forward, we propose to develop a culturally acceptable intervention tool that accommodates adolescents’ perspectives and psychosocial context.

## Introduction

Psychological stress is an unpleasant subjective feeling perceived when an individual’s situational demands exceed their adaptive capacity ^1^. Persistent stress is linked to an increased risk of cardiovascular disease, diabetes mellitus and other adult non-communicable diseases ^2^. Psychological stress can also precipitate adverse mental health outcomes such as anxiety, depression and suicidal ideations ^3^.

Adolescence is a period of increased stress perception and reactivity ^4^. Various studies suggest rising prevalence of stress among adolescents and its increasing influence on adolescent health ^5, 6^. A rapid change in physical, psychological, physiological and social aspects during transitional adolescent phase may heighten stress reactivity ^4^. Abnormalities due to stress in physiology and psychological status during this transitional phase is likely to set lifestyle and risk trajectories in motion for future disease development. Intervention measures to address exaggerated perception of stress during this sensitive period may benefit future health.

One of the key considerations for developing interventions is to assess the need for this among adolescents through a deeper understanding of their perception of stress and stressors, and their in-built coping abilities. A “person-based approach” to intervention, where adolescents’ perspectives and psychosocial context of the intervention are understood, and accommodated in a systematic manner through qualitative research, is thought to strengthen the evidence-base and acceptability of interventions ^7^. SRAVANA (Stress Responses in Adolescence and Vulnerability to Adult Non-communicable disease) is a multifaceted study to examine the life course biological and psychological factors that determine stress among adolescents and to develop complex interventions for its management ^8^. As part of this initiative we undertook a qualitative study in a group of urban adolescents. The objectives were to explore adolescents’ perception of stress and stressors in their daily life, understand the strategies by which they cope with daily stresses, and identify the areas where they seek further support.

## Methods

### Study setting

The study was undertaken among urban-dwelling adolescents in Mysore in southern India. Mysore is a densely populated city of over 1 million residents, living mainly in individual homes and a smaller proportion of 3–4 storey apartment buildings. The city consists of several old neighbourhoods, many of which were established more than 150 years ago, and new extensions to accommodate its growing population. About 20% of the population live below the poverty line, while the rest can be classified as middle class. Mysore residents are engaged in both skilled and unskilled labour.

### Ethics approval

The study was approved by the Ethics Committee of the CSI Holdsworth Memorial Hospital, Mysore (June 2017; no. CSIHMH/ERU2017/1). The committee membership follows Indian Council of Medical Research guidelines and is composed of a total of nine members (2 clinicians, 3 scientists, 1 legal expert, 1 social scientist/ spiritual leader, 1 academician and 1 lay person).

### Participants and recruitment

Study participants were purposively recruited until data saturation was achieved through the interviews; the final sample consisted of 10 older adolescents (17–25 years of age) and six younger adolescents (12–15 years), with an equal number of boys and girls in each group. The older adolescents were recruited via telephone call from the Parthenon Cohort, an ongoing prospective birth cohort study at Holdsworth Memorial Hospital (HMH) in Mysore to examine the developmental origins of adult disease ^9^. The well-established relationships developed during follow-up of this cohort since birth, were helpful in creating an environment of trust for them to open up and share details about their lives. The younger adolescents were recruited by word of mouth from among those known to existing contacts, which included collaborators of previous research projects and acquaintances and friends of former and current employees of our Research Unit. Adolescents who were interested in participating then contacted the research team directly.

All study procedures were approved by the Institutional Review Board of HMH. After explaining the goals and objectives of the research, written informed consent was obtained from older adolescents and the parents/ caregivers of younger adolescents; younger adolescents gave their own written informed assent for participation.

### Data collection

The present study comprised 16 in-depth interviews (IDI) guided by a semi-structured interview schedule ( Table 1). Because of the potentially sensitive nature of the data, IDIs were chosen as the most appropriate method. Furthermore IDI’s are more likely to give insights into adolescents’ experiences in a detailed manner that would not be feasible with a structured questionnaire. The semi-structured interview comprised open- ended items to facilitate in-depth exploration related to the following:

**Table 1.** Semi-structured interview schedule used in the study.

In-depth Interview: topic guide
**What makes adolescents stressed?** **A.** What do you understand about stress? **B.** What situations create stress in your everyday life? **C.** What factors in everyday life may increase young people’s stress levels? **D.** What do you think about stress in other people of your age?
**What effects does stress have on adolescent’s daily life?** **A.** When you are stressed, how do you feel? Physical; psychological **B.** What effect does it have on your everyday life? Social life; academic life; relationships **C.** How do your peers and family react when you are stressed? **D.** According to your observations, how do people of your age behave when they are stressed?
**How do adolescents cope with stress?** **A.** How do you manage stress? **B.** What measures do you take to reduce the bad effects of stress? **C.** Who do you approach when stressed? **D.** What help do you seek from family and friends and how do they help? **E.** What is your family and friends’ approach to stress compared to you? **F.** What activities help reduce your stress?
**Whether it helps to get extra support from others?** **A.** What extra help from others do you think is useful to reduce stress? Family; friends **B.** What help can we as a hospital provide you to help you manage your stress? **C.** How can your school/college/workplace help in this?

1. What makes adolescents stressed?
2. What effect does stress have on adolescents’ daily life?
3. How do they cope with stress?
4. Does it help to get extra support from others?

This guide was developed by the Mysore research team (GVK, SVJ, MK and KK) and qualitative researchers at the University of Southampton (MB and CHDF). It was pilot tested with two IDIs which were included in subsequent data analysis. No changes were implemented as a result of pilot testing. The interviews were conducted between March 2018 and March 2020.

The questions and probes in the guide were asked in a sequence to promote a logical and smooth flow of discussion. The interviews lasted between 17 and 36 minutes. Recruitment of new participants was stopped when we arrived at the stage of data saturation when new themes stopped emerging.

All IDIs, except one which was conducted at a children’s hostel, were carried out at the HMH Research Unit, and by one male and two female investigators who were medical doctors and doctorate holders, and trained by experts in qualitative research methods. The older adolescents were known to the interviewers as long-term participants of the Parthenon cohort. Younger participants were not acquainted with the interviewers. The interviews were conducted on a one-to-one basis, in a separate, sound-proof room. Each session started with an ice breaker question. Depending on the participants’ preference, IDIs were conducted in either English or the local language, Kannada, or a mixture of the two, which is a common way of communication among Mysore residents. 

### Transcription and translation

All IDIs were audio recorded and transcribed verbatim; participants were anonymised by replacing names with codes. Whenever Kannada was used, audio files were transcribed into English directly because the transcribers were fluent in both languages. The interviews thus transcribed were checked back against the original audio files to ensure accuracy (by SVJ).

### Thematic analysis

The qualitative data in this study was analysed using the approach of Braun and Clarke (2006) ^10^. All the IDI transcripts were coded and analysed manually coded by TKN, SVJ and GVK; we did not use a qualitative research software for analysis. An initial coding framework (with a list of codes) was developed. As new codes emerged, they were incorporated into the coding framework. Finally, codes that were related to each other were categorized together under higher order codes to identify the main themes that emerged from the data set. Issues that were mentioned only once or by a single participant were included as additional, standalone points of view. Direct quotes from the IDIs were selected and used in the manuscript to illustrate specific issues or pieces of information that the participant wished to convey. Reporting of the study findings followed consolidated criteria for reporting qualitative research (COREQ) guidelines ^11^.

## Results

Among the ten older adolescents (17–25 years; mean [SD]: 19.9 [2.3] years) two girls were married and living with their spouse and his immediate family; 6 were still studying, and 3 were in paid jobs including one boy who was also attending university. All the six younger adolescents (12–15 years; 13.7 [1.0] years) were in school; 5 of them were living with their family, while one was under the care of a hostel warden.

Data from the IDI’s are presented below as themes and subthemes under three main headings related to stress and coping strategies.

### Theme 1. Stressors and stress

In general adolescents perceived that they experience stress in their daily life. Despite viewing stress as a part of life, there was a definite indication of its undesirable impact in their lives. They identified a range of stressors which are categorised as subthemes presented below. 

***Family is the main thing*.** Family aspects were the most prominently indicated stressors especially among older adolescents. The adolescents felt that family pressures and/or conflicts triggered a range of uncomfortable emotions in them.

*Pressure from the family:* The adolescents felt that being forced, either openly or implicitly, to take part in a range of activities and responsibilities was indeed stressful. These included direct pressure to socialise within extended families, expectations to conform to the family structure and its values, and a felt obligation to maintain family bonds and take general responsibility of the family.

*“How to manage family in the future…that’s also a stress, and besides that younger and older sisters’ marriage, their marriages how to do, where to do…that is also a stress”* (Participant # 8, Older adolescent, Male)

In younger adolescents, parental pressure for good academic performance was the major stressor.

*“ They’ll (parents) give a lot of tension - You HAVE to study like this, you HAVE to score this percentage; they’ll put a lot of pressure on the children. That creates tension for us”* (Participant # 11, Younger adolescent, Female)

*Family conflicts:* There was an underlying sense of conflict with family members or family circumstances among several adolescents. This was particularly highlighted by the two married participants (both girls). They felt that being chastised by the elders in the spouse’s family was an everyday background stress in their lives which led to constant displeasure.

*“Oh the usual things...like when someone scolds....like if I’ve made a mistake, done something wrong...it’s the usual....I feel bad and it causes daily stress. I’ll feel bad because I’ve done that (something wrong)”* (Participant #5, Older Adolescent, Female)

A few participants indirectly suggested that a lack of happiness in the family could be stressful. Stress was also triggered by a feeling of being neglected by their parents. One particular participant also mentioned that they perceived an abusive family environment as a stressor.

***Academics and workplace*.** Pressure to perform well academically featured repeatedly as one of the major stressors, mainly in younger adolescents. Stress was triggered not only from family and teachers, but their own fears for their future.

*“...poor thing right, kids? We also will become so stressed right? And if we don’t score well, what will happen to us in the future”* (Participant #11, Younger adolescent, Female)

Stress was also created by a non-conducive and conflict-ridden academic environment, or work environment in the case of some older adolescents. The adolescents felt greatly stressed by a poorly scheduled school curriculum leading to time pressure, an inadequate school infrastructure with the lack of essential facilities such as a cafeteria, or vindictive and hostile teachers.

*“Some teachers don’t particularly like some students.. and .. I don’t know.. some of them have a grudge against OUR class.. so that irritates me…..”* (Participant # 16, Younger adolescent, Female)

***Peers*.** Peer-induced stresses were not of major concern among our participants. A few older adolescents felt that a sense of inferiority or fear of missing out on good things when comparing themselves with their peers’ situation was stressful. Issues around romantic relationships, including pressure of impressing their boyfriends/ girlfriends or painful breakups were also thought to cause stress in some older adolescents. Two younger adolescents mentioned that minor fights with other schoolmates were stressful as it might lead to castigation from parents or teachers. In a few younger adolescents, comparing their academic scores with those of their peers was a source of stress. One adolescent even expressed that it is unfair for them to get lower marks despite putting in similar efforts to the top scorer.

*“It’s because both should have equal.... The thing is anyone would like to be at the top of the class, right? ....if one is not the topper... Just like that, both [of us] should get the same marks.... After putting in hard work and studying, if [we] don’t get marks then we feel …somewhat”* (Participant # 13, Younger adolescent, Male)

***Money and social position*.** Socio-economic issues appeared to be a source of stress only in older adolescents in our study. The most common feeling was the lack of nicer things that others had and a feeling of inferiority. For some, lack of money, and the resultant discontent over unmet desires for a better life was a stressor.

*“My father’s...my father has still not built a separate house...what I mean is, we have been living in the same place for the past 18 years…… Our father loses a lot of money. Instead of losing money, it would be good if he builds a house, or if he buys things for our home”* (Participant # 1, Older adolescent, Female)

***Gender dynamics*.** Two out of the three young adolescent girls in the study voluntarily highlighted specific issues involving boys. In both instances the participants felt that the situation was highly stressful. One girl expressed that girls being friendly with boys in the class was not approved of by their teachers, which resulted in them being unpleasant to her. This girl, who was visibly upset, said that this ‘annoyed’ her. Another girl said that she feels uncomfortable because boys stare at her while she walks to after-school tuitions. She said that such situations were highly upsetting and impeded her daily life activities.

*“boys will be standing and I feel somewhat to go. That’s all ...They will be staring at us when we go... So , I feel somewhat! If they are standing I feel like why to go now? I don’t know, but I feel bad....They are standing there, don’t want to go to the tuition....No, near my house.. I feel bad... They will see me.”* (Participant # 14, Younger adolescent, Female)

### Theme 2. Stress expression

Stress was reactively expressed through various emotions as indicated by the adolescents. There was a clear distinction between the younger and older participants in these reactions. Anger was the recurring feeling amongst most of the older adolescents. One participant expressed that they had violent thoughts about ‘scolding’, ‘beating’ and even ‘killing’ the person who caused the stress; a few said that the anger resulted in aggressive behaviours towards unsuspecting family members.

*“Like someone would have scolded me...I would have taken that anger out on someone else...like that...or take it out on the baby (laughs slightly)”* (Participant # 5, Older adolescent, Female)

Stress appeared to present as fear in younger interviewees, mostly due to the worry arising out of not doing an expected task like ‘homework’ (fear of teachers) or getting low marks in the exams (fear of parents).

*“So their parents…..they yell at them or make them feel bad. And a lot of my friends actually cry in school when they score less marks because they’re worried about what will happen at home”* (Participant # 16, Younger adolescent, Female)

Other common reactionary emotions were sadness, feeling hurt, annoyance or feeling guilty. 

### Theme 3. Coping and seeking help

Generally the participants were insightful that being stressed was not good for them, and that they wanted relief from it almost immediately. This was reflected in the process by which they chose their coping strategies. We identified two distinct actions (see below), which either alone or in tandem brought prompt relief from stress.

***Distancing from the situation causing stress*.** The first [instinctive] response towards coping involved adolescents appearing to distance themselves from the stressful situation, either physically by taking time off, staying alone, or getting out of that place; or emotionally, withdrawing from the source of stress by remaining silent. This was most common among older adolescents, and was often effective in relieving the stress.

*“I don’t get stressed....as soon as I get angry or whatever, I’ll fall silent. I won’t talk to anyone....by doing that I will bring my anger under control by myself.....”* (Participant # 2, Older adolescent, Male)

***Reflection*.** Both younger and older adolescents indicated that stress was frequently followed by reflective, positive thoughts. They were usually aware of their emotions, tried to understand others’ points of view and the circumstance, accepted the situation, and generally put things into perspective.

*“....… we shouldn’t take much stress, shouldn’t take too much tension…. what will happen it will happen, we cannot do anything about it… so what is going on we should be happy with that, without thinking too much about what should happen and shouldn’t happen… what might happen will anyway happen, so just focus on our goals and go on”* (Participant # 8, Older adolescent, Male)

***Specific coping strategies*.** Some adolescents also mentioned that following the processes of distancing and reflection in sequence also led to choosing a specific coping strategy that worked best for them in that situation. Some of these were related to specific actions to calm the mind, including meditation and taking rest. Others were distraction activities, including sports, outdoor walks, long drives, creative art, video games, watching favourite television shows and eating ( *“Tummy full, no anger”*; Participant # 4, Older adolescent, Female). A few adolescents relied on avoidance strategies from stressful events or people. Two of the younger adolescents also found help in spirituality.

*“I’ll go to...I’ll go to the ‘ **devara mane’** (prayer room)...I’ll look at God and start (my work). If there is any tension and I am not able to tell Mother, I’ll go to the devara mane’, tell it in front of **God** and I will just calm myself.”* (Participant # 11, Younger adolescent, Female)

Sharing their worries with others acted as an effective coping strategy in some of them, though this was not the first choice in older adolescents. Sharing was mainly with friends in the older adolescents, and both friends and family in younger participants. Both the married girls confided and regularly discussed their issues with their spouse, which they found was helpful.

*Risk behaviour:* A few older participants suggested that either they or their peers may seek risky or harmful strategies to get immediate relief from stress. These were mainly related to the consumption of alcohol and smoking. One adolescent also said that fast driving is another risky coping strategy among their peers. All of these adolescents, except one, suggested that these behaviours were noticed among their peers, but not in themselves. The adolescents thought that this may lead to an addictive behaviour on repeated stress.

*“You know the thing is when we are very stressed out and people go and do cigarettes no, uh it feels...we feel relief, ok? So what happens next time we feel stressed.. uh.. one more cigarette.. one more cigarette.. and it goes on."* (Participant # 4, Older adolescent, Female)

Coping strategies usually brought relief from stress which participants described as happiness, calmness, peace, relaxation, or a sense of being free and refreshed.

***Seeking help*.** Generally, all participants indicated that they seek practical solutions to their problems from adults. Adolescents felt that the adults should listen to their worries without judgement. This sentiment was particularly highlighted by one girl who preferred to share her stresses with her doll because *“it did not talk back”* (Participant # 10, Older adolescent, Female). A few adolescents sought a regular visit to a youth centre where they can relax through activities or discuss their issues and come up with solutions. One participant felt that talking to a person close to their age was more helpful than discussing their issues with an older adult. Another boy felt that adolescents need personal space and time to recover from stress.

*“… if someone is stressed, they should simply be left alone to relax for 10–15 minutes. No one should try to talk to them…I feel that would give them relief.”* (Participant # 2, Older adolescent, Male)

## Discussion

This study aimed at an in-depth understanding of stress and stressors among adolescents, we found that youngsters, irrespective of age and gender, perceived stress in daily life. Family and academic pressures were considered the major stressors. Adolescents seemed to have efficient coping strategies and support for the prompt relief from stress. Additional support was mainly sought for practical solutions to the stressful situations.

Adolescence is described as a period of ‘storm and stress’, owing to intense stress perception and reactivity characteristic of this age ^4^. Widespread neuro-cognitive and behavioural changes and changing patterns of social interactions are thought to underlie this transitional phase. Adolescents form about 20% of the population in India, one of the largest percentages in the world ^12^. Recent reports of increasing mental health issues, including depression and suicidal tendencies among youth may suggest an increasing prevalence or perception of stress ^6, 13^. Though nationally representative data on adolescent stress is not available, it can be anecdotally assumed that some amount of stress is universal in today’s adolescents. Structured tools designed for specific situations and population may not identify small stressors and stresses which may have huge implications for their well-being.

Our study is one of few to use qualitative methods to understand the concerns around stress among adolescents in India, and the first to use in-depth interviews as far as we know. We found that stress is ubiquitous in adolescents, irrespective of age. The triggers for stress ranged from minor conflicts to serious issues around financial responsibilities and abuse. Family pressure was one of the major stressors for adolescents. Previously, other qualitative and quantitative studies have identified family-related stress perception among adolescents ^14– 16^. This was thought to result from a perception of challenges around establishing an independent social identity ^16^. The conflict between a traditional family environment instructing what adolescents should do, and adolescents’ aspirations for autonomy may bring out tensions within the family. Academic pressure appeared to be another major trigger for stress. Several studies have highlighted that children and adolescents in India are under high pressure to perform well in academics ^15, 17– 19^. which is a major deciding factor for their future career options in a highly competitive and stressful environment of limited opportunities ^20^. This has been shown to influence several aspects of their lives including lifestyle habits such as diet and physical activity ^21, 22^, apart from increased prevalence of anxiety and depression ^17– 19^. Interestingly, peer-issues, which feature commonly in other studies was not a major determinant of stress according to our participants.

Notwithstanding these stresses, our youth appeared to have effective coping methods in place. Strategies to relieve stress were varied – distraction activities, avoidance, and calming methods were some of the methods employed, which are well established coping strategies reported in other studies from high-income countries ^23^. However, the most common and effective strategy seemed to be cognitive. Putting things into perspective was employed by both younger and older adolescents. They seemed to understand the futility of continued stress perception, as well as its adverse implications for their well-being. Similar coping methods have been observed in other studies in India ^12, 14– 16^.

### Strengths and limitations

The main strength of this study is that we conducted in-depth interviews rather than group discussions, which allowed youngsters to divulge sensitive information related to their stress in the privacy of a closed session with a single interviewer. Also, including both male and female adolescents in our sample helped to identify a large number of stressors and coping strategies that were both similar and disparate between the genders. Further, the participants were from diverse backgrounds - recruiting both younger adolescents who were full-time students, and older adolescents who were students, paid employees, and/or married, provided a broader understanding of stress in these age groups, allowing us to capture qualitative data that was rich. Another strength was the provision for open ended questions allowing for in-depth inquiry when indicated.

One possible limitation was uneven numbers of younger and older adolescents in the analysis, which may have limited a meaningful comparison of the two groups. However, our study was not designed *a priori* to test these differences, but we felt that the observed differences by age were important to report. Also, a female researcher interviewed most of the adolescent boys, and due to the nature of the research topic, boys at this stage of life might not have felt comfortable enough to share certain types of information, although none of them refused to participate and reported feeling inhibited during the interview in the feedback. Another limitation may be a smaller sample size. However, we continued the participant recruitment until the data saturation was perceived, and therefore, surplus numbers were less likely to have discovered additional concepts. Moreover, our sample size conforms to the expected numbers for individual interview designs in other qualitative studies ^24^.

In conclusion, developing interventions to manage stress among adolescents requires a better understanding of their own perception of the need and the method best suited for their situation. In this respect, our study suggests that measures that stimulate cognitive appraisal and understanding of the stressful situation may be more effective in developing resilience to stress among adolescents in this urban setting. This may help to change the lifestyle trajectories of those adolescents who may resort to risk behaviours as an escape from stress. We propose that a physical or virtual youth platform that offers personal space and an outlet for adolescents to express their stresses may help mitigate the long-term effects of every day stresses in this crucial life stage. Our future efforts are aimed at developing interventions incorporating these principles.

## Data availability

The study data are not freely available due to ethical and security considerations. Anonymised interview transcripts data are available on reasonable request subject to Health Ministry Screening Committee (HMSC), India and Institutional Ethics Committee approval. For further information contact the corresponding author: Dr. GV Krishnaveni ( gv.krishnaveni@gmail.com).
