# Supplementary material for: What stresses adolescents? A qualitative study on perceptions of stress, stressors and coping mechanisms among urban adolescents in India
Source: Wellcome Open Res. 2021 May 11;6:106. [Version 1] doi: 10.12688/wellcomeopenres.16818.1 (PMC8170530; doi:10.12688/wellcomeopenres.16818.1)
Supplement: Peer review history

# Peer review: Reviewer response for version 1

**DOI:** 10.21956/wellcomeopenres.18552.r43844

**Published:** 2021-06-01

**Authors:** Shilpa Aggarwal

**Affiliations:** 1 Faculty of Health, School of Medicine, Deakin University, Geelong, Vic, Australia; 2 Public Health Foundation of India, New Delhi, Delhi, India

This is an important qualitative study to explore the triggers for stress and coping in Indian adolescents that could inform an intervention. Some suggestions to improve the clarity of the report for the readers.

**Introduction:**

 It is important to highlight that the experience of stress is universal and is a continuum. This becomes important, especially while talking about the interventions. Are the authors referring to universal interventions for adolescents due to their “increased stress perception” as suggested in the first sentence of the second paragraph or for those who are experiencing more stress as compared to their peers? Information about the type of intervention authors are recommending or aiming to develop would help the readers put the current work in context. The statement will have to be referenced. The same comment is applicable to the first statement in paragraph 3.

**Methods:**

 In the methods section, some details about the adolescent population in Mysore (as the sample is selected from this pool) would be useful (e.g. total number of adolescents with the age range, number of school going adolescents and if there is any data available regarding the number of married adolescents- gender based differences in social demands and expectations due to marriage could result in excessive stress in married females).

 In the methods section, the inclusion criteria (and exclusion criteria, if any) need to be included- if being an adolescent and residing in Mysore was sufficient to be included in the study or if self-reported stress was a prerequisite. If so, was any measure of stress used for assessment.

 Some information about how the age groups were stratified for the study and if there is any specific reason for excluding 16 year-old adolescents. A reference to the selection of older adolescent participants from the Parthenon cohort and well established relationship with this cohort is made under the participants and recruitment section, 1 ^st^ paragraph. Did the researchers use any particular criteria while deciding which adolescents to contact for the study (sufficient information regarding inclusion criteria would address this comment).

 Including details of the participants in a tabular form including their age, gender, whether they were in school/college/employed/homemakers and if they belonged to the Parthenon cohort/selected by any other means would make it more informative.

 Also, the selection of adolescents and interviews took 2 years. It might be useful for the readers to know of any challenges during recruitment leading to the delay as the selection of the majority of adolescents (older adolescents, 10 in number) was done from an already engaged Partethon sample.

 Although the authors have included a definition of psychological stress as “an unpleasant subjective feeling perceived when an individual’s situational demands exceed their adaptive capacity”, the interview guide and the probes don’t suggest any exploration of feeling overwhelmed or it exceeding their adaptive capacity. An explanation in this regard would be helpful to understand how the construct of stress used in this study.

**Results:**

 For two older adolescent girls who were married, it might be useful to include their employment status (if they were homemakers or employed in a paid job).

 The authors have used Braun and Clarke approach ^1^ of thematic analysis. Some information about the paradigm used within this approach while conducting the analysis will be helpful to understand the findings better. In theme 2, the expression of stress has been mainly covered in the way it is experienced emotionally. Were there any behavioural manifestations identified by the authors during the interviews? If so, including it as a subtheme would be useful. The authors have included vignettes from the interviews. The language of the vignettes appears to be a literal translation from the language in which the interviews were conducted. Making sure that the vignettes are grammatically correct and complete would help the readers to understand the meaning better.

**Discussion:**

 Kindly reframe the sentence in the third paragraph “Previously, other qualitative and quantitative studies have identified family-related stress perception among adolescents.” as the meaning is not very clear.

 Elaborating on the meaning of the findings and how this would translate into developing an intervention would be useful.

 There are sentences added in the conclusion section about how the study findings would help in the intervention development. It is not very clear how the conclusion (appended below) has been derived from the findings and discussion. The meaning of the statements is not very clear either- elaborating upon the findings in the discussion (with a view to reach this conclusion) and reframing the sentences to improve clarity would be helpful.

 “In conclusion, developing interventions to manage stress among adolescents requires a better understanding of their own perception of the need and the method best suited for their situation. In this respect, our study suggests that measures that stimulate cognitive appraisal and understanding of the stressful situation may be more effective in developing resilience to stress among adolescents in this urban setting.”

Is the work clearly and accurately presented and does it cite the current literature?

Partly

If applicable, is the statistical analysis and its interpretation appropriate?

Partly

Are all the source data underlying the results available to ensure full reproducibility?

Yes

Is the study design appropriate and is the work technically sound?

Yes

Are the conclusions drawn adequately supported by the results?

Partly

Are sufficient details of methods and analysis provided to allow replication by others?

Yes

Reviewer Expertise:

Child and Youth Mental Health, Global Mental Health

I confirm that I have read this submission and believe that I have an appropriate level of expertise to confirm that it is of an acceptable scientific standard.

## References

[1] : An Arts on Prescription Programme: Perspectives of the Cultural Institutions. *Community Ment Health J*.56(8) : 10.1007/s10597-020-00591-x 1473-1479 10.1007/s10597-020-00591-x 32100154

# Peer review: Reviewer response for version 1

**DOI:** 10.21956/wellcomeopenres.18552.r43847

**Published:** 2021-05-24

**Authors:** Abhijit Nadkarni, Soumya Singh

**Affiliations:** 1 Centre for Global Mental Health, Department of Population Health, London School of Hygiene & Tropical Medicine, London, UK; 2 Sangath, Porvorim, Goa, India

**Summary:**

 The article explores the perceptions and experiences of stress, sources of stress and coping strategies among young people aged 10-25 years in Mysore, India. The study uses a qualitative approach to understand context-specific nuances of stress and coping among adolescents, with the aim to use these findings to develop an intervention for stress management. They report differentiated findings on the sources of stress for younger and older adolescents, and coping strategies that help adolescents deal with them effectively. This formative research is important in its contribution to an in-depth understanding of stress and highlights the need for adolescent mental health interventions in India.

**Major comments:**

1. Clarify the type of intervention that you aim to build i.e. stress prevention, stress management, etc.
2. The introduction section does not emphasise the scope of the problem. It would be useful to add statistics from studies conducted on adolescent stress (global > low- and middle-income countries >India).
3. In the methods section (‘Study setting’), elaborate on information about adolescents living in Mysore instead of generic information about Mysore.
4. Reference the statement about IDIs being the best tool to collect sensitive data.
5. There’s lack of clarity in “It was pilot tested with two IDIs which were included in subsequent data analysis. No changes were implemented as a result of pilot testing.” Please clarify why no changes were implemented.
6. Suggest explaining why qualitative research software was not used for analysis.
7. Suggest presenting demographic data in a table format; It is not required to share the marital status of women if the same is not done for adolescents boys/young men.
8. Maintain consistency in reporting findings i.e all participants>older adolescents>younger adolescents OR younger adolescents>older adolescents>all participants.
9. Discussion appears to focus more on existing data than the study findings. It would be helpful to expand on the study findings.
10. Use consistent terminology to describe the study population. Currently the article uses different terminology i.e. youngsters, young people, adolescents.
11. Use consistent terminology for the same construct. For example, abstract mentions self-comforting methods as a coping strategy whereas discussion mentions ‘calming methods’ as a coping strategy

**Minor comments:**

 Abstract:

1. Replace volunteers with participants in “We recruited 16 volunteers in Mysore”

1. In “India including 6 younger (12-15 years; 3 girls) and 10 older adolescents/ young adults (17-25 years; 5 girls)”, remove the number of girls from the description unless mentioned for any specific reason.
2. In “Older adolescents were recruited from ongoing birth cohort study”, name the ongoing birth cohort study.
3. In “The interviews were analysed to derive themes and emerging constructs related to stress and coping strategies”, mention the framework used for analyses of the data.
4. “Adolescents generally perceived stress in their daily lives”, the statement is vague and could be rephrased as “Adolescents generally experienced stress in their daily lives”.
5. It is unclear whether adolescents reported ‘robust’ coping strategies in “Adolescents reported that they had robust coping strategies” or whether the coping strategies were reported as ‘robust’ by the researchers.

Introduction:

1. In “Abnormalities due to stress in physiology and psychological status during this transitional phase is likely to set lifestyle and risk trajectories in motion for future disease development”,  remove ‘lifestyle and’.
2. In “One of the key considerations for developing intervention”, change ‘for’ to ‘in’.
3. It is unclear what ‘this’ means in “One of the key considerations for developing interventions is to assess the need for this among adolescents through a deeper understanding of their perception of stress and stressors, and their in-built coping abilities.”
4. Introduce SRAVANA in the abstract as it may seem abrupt when mentioned only later in the introduction.
5. Change “The objectives were to explore adolescents’ perception of stress and stressors in their daily life, understand the strategies by which they cope with daily stresses, and identify the areas where they seek further support” to “The study objectives were to explore adolescents’ perception of stress and stressors in their daily life, understand the strategies they use to cope with daily stresses, and identify the areas where they seek further support”

Methods:

 Participants and recruitment:

1. Suggest changing “The younger adolescents were recruited by word of mouth from among those known to existing contacts, which included collaborators of previous research projects and acquaintances and friends of former and current employees of our Research Unit. Adolescents who were interested in participating then contacted the research team directly” to “ Younger adolescents were recruited by word-of-mouth from existing networks including research collaborators of previous projects and community networks of former and current employees of our research unit. Interested adolescents directly contacted the research team to participate in the study”.
2. Suggest removing “After explaining the goals and objectives of the research”, as this is standard procedure that doesn't necessarily need to be mentioned.

Data collection:

  

1. Suggest changing “the present study comprised 16 in-depth interviews (IDI) guided by a semi-structured interview schedule “ to “We conducted 16 in-depth interviews using a semi-structured interview schedule”; Also suggest replacing schedule with a guide.
2. Mention ‘semi-structured IDIs’ and not just IDIs in the following statement “Because of the potentially sensitive nature of the data, IDIs were chosen as the most appropriate method. Furthermore, IDI’s are more likely to give insights into adolescents’ experiences in a detailed manner that would not be feasible with a structured questionnaire.”; Remove ‘furthermore’. Suggest adding this because you may compare semi-structured interviews with structured interviews but not IDIs with structured interviews as IDIs may also be structured.
3. Suggest removing initials in “This guide was developed by the Mysore research team (GVK, SVJ, MK and KK) and qualitative researchers at the University of Southampton (MB and CHDF).”
4. Suggest replacing “The questions and probes in the guide were asked in a sequence to promote a logical and smooth flow of discussion” with “The questions and probes in the guide were asked sequentially to promote a smooth flow to the interview”.
5. Suggest changing “Recruitment of new participants was stopped when we arrived at the stage of data saturation when new themes stopped emerging” to “Recruitment of new participants was stopped when data saturation was reached and new themes stopped emerging”
6. Break up the long sentence: “All IDIs, except one which was conducted at a children’s hostel, were carried out at the HMH Research Unit, and by one male and two female investigators who were medical doctors and doctorate holders, and trained by experts in qualitative research methods.”

Transcription and translation:

1. In “All IDIs were audio recorded and transcribed verbatim; participants were anonymised by replacing names with codes”, I suggest replacing participants with interviews.
2. Remove initials of the transcriber

Thematic analysis:

1. The following statement is grammatically incorrect: “All the IDI transcripts were coded and analysed manually coded by TKN, SVJ and GVK”; Remove initials.
2. In “Direct quotes from the IDIs were selected”, remove ‘direct’.
3. Suggest changing “Direct quotes from the IDIs were selected and used in the manuscript to illustrate specific issues or pieces of information that the participant wished to convey” as the illustrations are not about what the participant wished to convey but what the researcher aimed to ask to fulfill research objectives. A possible replacement would be, “Illustrative quotes from the IDIs were selected and used in the manuscript to highlight specific issues or pieces of information on adolescents’ perceptions of stress, stressors and coping strategies”.

Result:

1. Suggest replacing “Data from the IDI’s are presented below as themes and subthemes under three main headings related to stress and coping strategies” with “Major themes and subthemes from the study are presented below”

Themes and Subthemes:

 Theme 1: Stressors and stress

1. Change heading to ‘stress and stressors’.
2. Add a comma after “In general adolescents perceived that they experience stress in their daily life”.
3. Replace ‘in’ their lives with ‘on’ their lives in the following: Despite viewing stress as a part of life, there was a definite indication of its undesirable impact in their lives.
4. Suggest changing “Family aspects were the most prominently indicated stressors especially among older adolescents” to “Family-related stressors were the most prominently indicated stressors especially among older adolescents”; The change is suggested because ‘family aspects’ as being a stressor appears to be vague.
5. Remove ‘the’ before adolescents in “Pressure from the family: The adolescents felt that being forced, either openly or implicitly”.
6. Change “socialise within extended families” to “socialise with extended families”.
7. Provide age next to illustrative quotes.
8. Suggest replacing “This was particularly highlighted by the two married participants (both girls)” with “This was particularly highlighted in interviews with two female participants who were married”.
9. It is unclear what ‘background stress’ means in “They felt that being chastised by the elders in the spouse’s family was an everyday background stress in their lives which led to constant displeasure”; suggest removing ‘background’ unless it is pertinent to the theme.
10. Replace “Stress was also created by” with “Stress was also experienced due to”.
11. Replace “time pressure” with “pressure to meet deadlines”.
12. Replace “our participants” with “study participants”.
13. Replace “Issues around romantic relationships, including the pressure of impressing their boyfriends/ girlfriends or painful breakups were also thought to cause stress in some older adolescents” with “Issues around romantic relationships, including pressure to impress their boyfriends/girlfriends or relationship breakups also caused stress in some older adolescents”.
14. In “Two younger adolescents mentioned that minor fights with other schoolmates were stressful as it might lead to castigation from parents or teachers”, clarify if the fights were reported as ‘minor’ by adolescents.
15. The following illustration seems incomplete: *“It’s because both should have equal.... The thing is anyone would like to be at the top of the class, right? ....if one is not the topper... Just like that, both [of us] should get the same marks.... After putting in hard work and studying, if [we] don’t get marks then we feel …somewhat”.*
16. Suggest using different terminology in “The most common feeling was the lack of nicer things..”.
17. Suggest replacing ‘gender dynamics’ with ‘gender-specific stressors’.
18. Remove voluntarily in the following statement: “Two out of the three young adolescent girls in the study voluntarily highlighted specific issues involving boys.”
19. Remove the following statement  “In both instances the participants felt that the situation was highly stressful.” as it is repetitive.

Theme 2:

1. Use ‘a’ instead of ‘Anger was the recurring feeling amongst most of the older adolescents.

Theme 3:

1. Change heading to ‘Coping and help-seeking’.
2. Suggest replacing insightful with aware in the following statement: “Generally the participants were insightful that being stressed was not good for them, and that they wanted relief from it almost immediately”.
3. Suggest replacing “The first [instinctive] response towards coping” with “The first [instinctive] coping response”.
4. The following statement is grammatically incorrect: “Some adolescents also mentioned that following the processes of distancing and reflection in sequence also led to choosing a specific coping strategy that worked best for them in that situation”. I suggest replacing it with “Some adolescents also mentioned that following the processes of distancing and reflection, they chose a specific coping strategy that worked best for them in that situation”.
5. Replace “distraction activities” with “engaging in distracting activities”.
6. Suggest replacing “also found help in spirituality.” With “also found spirituality to be helpful in coping with stress”.
7. Replace ‘in’ with ‘for’ in the following statement “Sharing their worries with others acted as an effective coping strategy in some of them”.
8. Replace “Sharing was mainly with friends in the older adolescents, and both friends and family in younger participants” with “Older adolescents shared their worries mainly with friends whereas younger participants shared them with both their friends and family”.
9. Suggest replacing “The adolescents thought that this may lead to an addictive behaviour on repeated stress” with “Adolescents thought that this may lead to addictive behaviours upon exposure to stress repeatedly”.
10. Replace ‘can’ with ‘could’ in the following statement: A few adolescents sought a regular visit to a youth centre where they can relax through activities or discuss their issues and come up with solutions.
11. The following statement is grammatically incorrect. I would suggest breaking it up into two statements and using consistent terminology i.e either young people, adolescents or youngsters and not all. *“This study aimed at an in-depth understanding of stress and stressors among adolescents, we found that youngsters, irrespective of age and gender, perceived stress in daily life”.*

Discussion:

1. “support for the prompt relief from stress” is not clearly indicated in the results section.
2. The statement “Structured tools designed for specific situations and population may not identify small stressors and stresses which may have huge implications for their well-being” implies that the study aims to focus on and understand ‘small’ stressors which is not true to the scope of the study. It could be replaced with “Structured tools designed for specific contexts and population may not efficiently identify context-specific, nuanced stressors and stresses which may have major implications for the well-being of adolescents”.
3. Part of the following section is a better fit for Introduction *“Adolescence is described as a period of ‘storm and stress’, owing to intense stress perception and reactivity characteristic of this age* *4* *. Widespread neuro-cognitive and behavioural changes and changing patterns of social interactions are thought to underlie this transitional phase. Adolescents form about 20% of the population in India, one of the largest percentages in the world* *12* *. Recent reports of increasing mental health issues, including depression and suicidal tendencies among youth may suggest an increasing prevalence or perception of stress* *6* *,* *13* *. Though nationally representative data on adolescent stress is not available, it can be anecdotally assumed that some amount of stress is universal in today’s adolescents. Structured tools designed for specific situations and population may not identify small stressors and stresses which may have huge implications for their well-being.”*
4. In “Our study is one of few to use qualitative methods to understand the concerns around stress among adolescents in India”, I suggest replacing ‘concerns around stress’ with ‘experience of stress’.
5. Remove full stop in the following “ *Several studies have highlighted that children and adolescents in India are under high pressure to perform well in academics* *15* *,* *17* *–* *19* *. which is a”.*

Strengths and limitations:

1. It is unclear what the following statement is trying to say and would require rewording “Also, a female researcher interviewed most of the adolescent boys, and due to the nature of the research topic, *boys at this stage of life* might not have felt comfortable enough to share certain types of information”.
2. Remove ‘to’ in the following statement: “This may help to change the lifestyle trajectories of those adolescents who may resort to risk behaviours as an escape from stress.”

Is the work clearly and accurately presented and does it cite the current literature?

Partly

If applicable, is the statistical analysis and its interpretation appropriate?

Yes

Are all the source data underlying the results available to ensure full reproducibility?

Yes

Is the study design appropriate and is the work technically sound?

Yes

Are the conclusions drawn adequately supported by the results?

Yes

Are sufficient details of methods and analysis provided to allow replication by others?

Yes

Reviewer Expertise:

Global mental health, complex intervention development and testing, addictions

We confirm that we have read this submission and believe that we have an appropriate level of expertise to confirm that it is of an acceptable scientific standard.
